# Bilateral Pregnancy Luteoma Presenting as Acute Abdomen in a Young Female: A Case Report

**DOI:** 10.7759/cureus.68852

**Published:** 2024-09-07

**Authors:** Rabia Nawaz, Zeeshan Ahmed, Hooriea Nauman, Muhammad F Nadeem, Abdul Haseeb Hasan, Muhammad Ali Abid

**Affiliations:** 1 Medicine, Mayo Hospital, Lahore, PAK; 2 Medicine and Surgery, King Edward Medical University, Lahore, PAK; 3 Medicine, Allama Iqbal Medical College, Lahore, PAK

**Keywords:** acute abdomen, bilateral pregnancy luteoma, gynaecology and obstetrics, luteoma, luteoma of pregnancy, non-neoplastic ovarian lesion, ovarian mass, pregnancy acute abdomen, pregnancy luteoma

## Abstract

Pregnancy luteoma (PL) is a rare, non-neoplastic ovarian lesion that can mimic malignant ovarian tumors, posing significant diagnostic challenges. PL typically presents as asymptomatic, unilateral, or bilateral ovarian masses and is often discovered incidentally. Its development is linked to hormonal fluctuations during pregnancy, particularly elevated human chorionic gonadotropin (hCG) levels. While PL generally resolves postpartum, complications such as torsion may necessitate surgical intervention. We report the case of a 23-year-old primigravida presenting with acute abdominal pain, vomiting, and abdominal distention at 13 weeks gestation. Imaging revealed large, bilateral multicystic ovarian masses. Elevated CA-125 levels raised suspicion for malignancy, leading to a laparotomy and bilateral oophorectomy. Histopathological analysis confirmed the diagnosis of pregnancy luteoma.

## Introduction

Pregnancy luteoma (PL) is a rare, non-neoplastic ovarian lesion [1,2]. Initially described by Sternberg and Barclay in 1966, PL remains a rare clinical entity, with less than 200 cases reported in the literature [1,3,4]. The pathogenesis of PL is believed to involve the overexpression of β-hCG, which induces uncontrolled proliferation of ovarian stromal cells, leading to the development of these lesions [1,5,6]. Although PLs are typically asymptomatic, they may occasionally present with symptoms such as acute abdominal pain, voice deepening, acne, hirsutism, and virilization [1,6,7]. These lesions can manifest as unilateral or bilateral solid ovarian masses, with diameters reaching up to 20 cm [6,7]. PLs are most frequently discovered incidentally during imaging or surgical procedures and generally resolve spontaneously postpartum [1,2,6,7]. However, there have been reports of recurrence in subsequent pregnancies [6].

The hormonal fluctuations inherent in pregnancy are considered a key factor in the development of PL. Furthermore, certain risk factors, such as advanced maternal age, polycystic ovarian syndrome (PCOS), and multiple gestations, have been identified as contributing to the likelihood of developing PL [2,6]. Accurate diagnosis of PL is crucial due to its potential to be mistaken for malignant ovarian tumors, which may lead to unnecessary oophorectomy and associated risks to both the mother and fetus [1,6,7].

This case report discusses a 23-year-old primigravida from an underprivileged region of Pakistan, with a body mass index (BMI) of 21, and no prior history of PCOS, who presented with bilateral pregnancy luteoma.

## Case presentation

A 23-year-old primigravida at 13 weeks of gestation presented with a one-week history of acute abdominal pain, accompanied by vomiting and abdominal distention that was disproportionately large for her gestational age. Physical examination revealed a hard, tender mass with irregular borders. Abdominopelvic ultrasound and magnetic resonance imaging (MRI) identified bilateral large, complex multicystic lesions, measuring 20 cm on the right and 19 cm on the left adnexa. Given the clinical suspicion of ovarian neoplasms, a comprehensive assessment was conducted, including tumor marker analysis and baseline laboratory investigations (Table 1). The markedly elevated CA-125 level further raised concerns regarding a potential ovarian malignancy.

**Table 1:** Summary of laboratory investigations Hb: Hemoglobin, TLC: Total Leukocyte Count, LFTs: Liver Function Tests, RFTs: Renal Function Tests, HBsAg: Hepatitis B Surface Antigen, Anti-HCV: Hepatitis C Virus Antibody, AFP: Alpha-Fetoprotein, CA-125: Cancer Antigen 125

Parameters	Results	Reference Ranges
Hb	9 g/dl	12-16 g/dL
Platelets	331 x10^9/L	150-450x10^9/L
TLC	7.7 x10^9/L	4.5-11x10^9/L
LFTs	Normal	-
RFTs	Normal	-
Urine Complete	Normal	-
HBsAg	Negative	-
Anti-HCV	Negative	-
AFP	6.4 ng/ml	0-10 ng/ml
CA-125	1309.6 units/ml	0-35 units/ml

**Figure 1:**
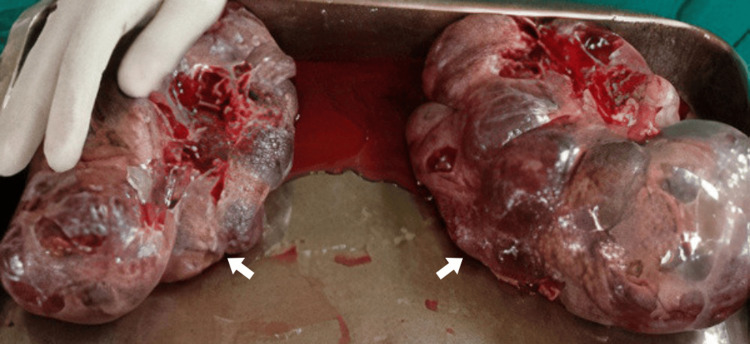
Gross anatomy of bilateral pregnancy luteoma showing soft, multicystic, and brownish masses (white arrows)

A staging computed tomography (CT) scan of the chest, abdomen, and pelvis was performed, which ruled out metastasis. However, during the workup, the patient underwent a miscarriage. A laparotomy was indicated due to the persistent and severe abdominal pain caused by the mass effect. During the procedure, a bilateral oophorectomy was performed, and omental and mesenteric samples were collected to exclude metastatic disease, which was confirmed negative. The gross examination of the resected ovarian masses revealed soft, multicystic, brownish lesions (Figure 1). The patient was subsequently initiated on lifelong hormone replacement therapy to manage the iatrogenic postmenopausal state resulting from the bilateral oophorectomy.

## Discussion

PL is a rare, benign, non-neoplastic ovarian lesion, first characterized by Sternberg in 1966 [1-3,8]. This condition, which typically emerges during pregnancy, can closely mimic the clinical and radiological features of malignant ovarian tumors, thereby presenting a significant diagnostic challenge [2,9]. Although PL is often discovered incidentally during cesarean sections or postpartum tubal ligations, it can sometimes be identified antenatally through imaging [2,6,8]. Despite its alarming presentation, PL is generally benign, with the masses typically regressing spontaneously within three months postpartum [2,9].

The exact etiology of PL remains unclear. However, it is hypothesized that the condition arises from pre-existing luteinized stromal cells that exhibit an exaggerated response to elevated β-hCG levels during pregnancy [4,8-10]. PL predominantly affects women in their third or fourth decade of life with increased prevalence in the African American population, and approximately one-third of cases are bilateral [7-9]. While many patients are asymptomatic, some may present with acute abdominal pain due to complications such as torsion [2,7,11]. In our case, the patient exhibited symptoms, including abdominal pain and tenderness, which are atypical for PL and more commonly associated with malignant ovarian masses. These symptoms are rarely reported in the literature, though there are documented cases, such as the one described in a case report, where the patient presented with massive ascites and elevated CA125 levels at 15 weeks gestation following ovulation induction therapy. Suspecting malignancy, a laparotomy was performed, ultimately revealing a PL [12]. It is imperative that PL be considered in the differential diagnosis of malignant ovarian cysts.

In approximately 25% of cases, PLs are hormonally active, leading to androgen secretion and subsequent maternal hirsutism and virilization [4]. In these cases, female fetuses may be affected, with up to half exhibiting clitoral enlargement and ambiguous genitalia, whereas male fetuses remain unaffected [4,13]. In our case, there were no signs of virilization, and therefore, hormonal studies were not conducted.

**Figure 2:**
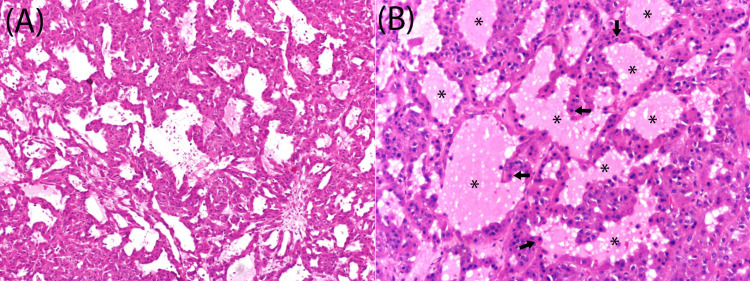
Microscopic appearance of pregnancy luteoma (A) showing masses of cells arranged in a follicular pattern (H&E X 100), (B) showing colloid-like material (black asterisks) filling the follicles that are lined by polygonal cells containing small, round nuclei and abundant eosinophilic cytoplasm (black arrows) (H&E X 400) Adapted with permission from Nanda et al. [9]

Macroscopically, cut sections of PLs present as well-circumscribed, solid, brownish-yellow masses, showing dark hemorrhagic foci, varying in size from microscopic to over 20 cm in diameter [1,7,9]. Microscopically, these lesions are characterized by cells arranged in trabecular or follicular patterns with accompanying stromal cell proliferation [1,9]. The cells typically have abundant eosinophilic and finely granular cytoplasm, with nuclei that may show mild pleomorphism (Figure 2) [2,9].

Certain conditions predispose women to the development of luteomas during pregnancy, with PCOS being a significant risk factor. The elevated hormonal levels associated with PCOS are believed to contribute to the formation of PLs [2,10,11]. Other risk factors include multiple pregnancies and advanced maternal age. Women with a history of luteomas in a previous pregnancy are at increased risk for recurrence [2,6].

The differential diagnosis of PL includes other solid ovarian neoplasms, such as granulosa cell tumors, thecomas, and Sertoli-Leydig cell tumors, which can be challenging to distinguish based on imaging alone [2,9]. MRI and CT imaging are superior to ultrasonography for visualizing the characteristic multiple nodules of PL. Once PL is suspected, detailed MRI and hormonal assays for testosterone and its derivatives are recommended [7].

When PL is suspected, clinical monitoring with postpartum follow-up is advised to avoid unnecessary surgical interventions, which could increase the risk of miscarriage or preterm delivery [5]. Although most cases regress spontaneously after childbirth, surgical intervention may be necessary in the presence of severe symptoms or complications such as torsion [5,10,11]. The management of PL should be tailored to the symptoms of the patient and overall condition. While some authors advocate for surgical treatment, typically, unilateral salpingo-oophorectomy, conservative management is preferred whenever possible, given the benign nature of the lesion [5,13].

## Conclusions

PL is a rare, benign ovarian lesion that poses significant diagnostic challenges due to its potential to mimic malignant ovarian tumors. While typically asymptomatic and often resolving spontaneously postpartum, PL can occasionally present with acute symptoms that necessitate surgical intervention. Accurate diagnosis and careful management are essential to avoid unnecessary procedures that may endanger both maternal and fetal health. Awareness of PL is crucial for clinicians, ensuring appropriate treatment and minimizing risks associated with misdiagnosis.

## References

[1] Rapisarda V, Pedalino F, Santonocito VC, Cavalli G, Zarbo G (2016). Luteoma of pregnancy presenting with severe maternal virilisation: a case report. Case Rep Obstet Gynecol.

[2] Verma V, Paul S, Chahal KS, Singh J (2016). Pregnancy luteoma. A rare case report. Int J Appl Basic Med Res.

[3] Sternberg WH, Barclay DL (1966). Luteoma of pregnancy. Am J Obstet Gynecol.

[4] Choi JR, Levine D, Finberg H (2000). Luteoma of pregnancy: sonographic findings in two cases. J Ultrasound Med.

[5] Masarie K, Katz V, Balderston K (2010). Pregnancy luteomas: clinical presentations and management strategies. Obstet Gynecol Surv.

[6] Phelan N, Conway GS (2011). Management of ovarian disease in pregnancy. Best Pract Res Clin Endocrinol Metab.

[7] Shang JH, Huang CX, Zheng Q, Feng JL, He K, Xie HN (2024). Imaging features, clinical characteristics and neonatal outcomes of pregnancy luteoma: a case series and literature review. Acta Obstet Gynecol Scand.

[8] Khurana A, OʼBoyle M (2017). Luteoma of pregnancy. Ultrasound Q.

[9] Nanda A, Gokhale UA, Pillai GR (2014). Bilateral pregnancy luteoma: a case report. Oman Med J.

[10] Agarwal I, Begum J, Singupuram NP (2022). Luteoma of pregnancy presenting as ruptured ectopic pregnancy: a case report. Cureus.

[11] Mvunta DH, Amiji F, Suleiman M (2021). Hirsutism caused by pregnancy luteoma in a low-resource setting: a case report and literature review. Case Rep Obstet Gynecol.

[12] Wang Y, Zhou F, Qin JL, Qian ZD, Huang LL (2015). Pregnancy luteoma followed with massive ascites and elevated CA125 after ovulation induction therapy: a case report and review of literatures. Int J Clin Exp Med.

[13] Cronjé HS (1984). Luteoma of pregnancy. S Afr Med J.

